# Mechanisms of acquired resistance to 2-(4-aminophenyl)benzothiazole (CJM 126, NSC 34445)

**DOI:** 10.1054/bjoc.2000.1231

**Published:** 2000-06-15

**Authors:** T D Bradshaw, M-S Chua, S Orr, C S Matthews, M F G Stevens

**Affiliations:** Cancer Research Laboratories, School of Pharmaceutical Sciences, University of Nottingham, Nottingham, NG7 2RD, UK

**Keywords:** 2-(4-aminophenyl)benzothiazole, MCF-7, acquired resistance

## Abstract

2-(4-aminophenyl)benzothiazole (CJM 126) elicits potent growth inhibition in human-derived breast carcinoma cell lines, including oestrogen receptor-positive (ER+) MCF-7^wt^cells. Analogues substituted in the 3′ position with I (DF 129), CH _3_(DF 203), or CI (DF 229) possess an extended profile of antitumour activity with remarkable selective activity in cell lines derived from solid tumours associated with poor prognosis, e.g. breast, ovarian, renal and colon. Growth inhibition occurs via unknown, possibly novel mechanism(s) of action. Two cell lines have been derived from sensitive MCF-7^wt^breast cancer cells (IC _50_ value < 0.001 μM) following long-term exposure to 10 nM or 10 μM CJM 126, MCF-7^10 μM 126^ and MCF-7^10 μM 126^respectively, which demonstrate acquired resistance to this agent (IC _50_> 30 μM) and cross-resistance to DF 129, DF 203 and DF 229. Sensitivity to tamoxifen, benzo[a]pyrene (BP), mitomyin C, doxorubicin and actinomycin D is retained. Resistance may, in part, be conferred by the constitutively increased expression of bcl-2 and p53 proteins detected in MCF-7^10 nM 126^and MCF-7^10 μM 126^lysates. Significantly decreased depletion of CJM 126 (30 μM) from nutrient medium of MCF-7^10 nM 126^cells was observed with predominantly cytoplasmic drug localization and negligible DNA strand breaks. *N* -acetyl transferase (NAT)1 and NAT2 proteins were expressed by all three MCF-7 sub-lines, but significantly higher expression of NAT2 was accompanied by enhanced acetylation efficacy in MCF-7^10 nM 126^cells. In contrast, CJM 126 (30 μM) was rapidly depleted from nutrient medium of MCF-7^10 μM 126^culture and accessed nuclei of these cells exerting damage to DNA. The major biotransformation product of CJM 126 in MCF-7^10 μM 126^cells was 2-(4-aminophenyl)-6-hydroxybenzothiazole (6-OH 126). This metabolite possessed no antitumour activity. Accordingly, in this sub-line, low constitutive expression and activity of cytochrome P450 (CYP) 1A1 was detected. © 2000 Cancer Research Campaign


*British Journal of Cancer* (2000) **83**(2), 270–277
© 2000 Cancer Research Campaign
doi: 10.1054/ bjoc.2000.1231, available online at http://www.idealibrary.com on IDEAL®

### Mechanisms of acquired resistance to 2-(4-aminophenyl)benzothiazole (CJM 126, NSC 34445)

**TD Bradshaw, M-S Chua, S Orr, CS Matthews and MFG Stevens**

Cancer Research Laboratories, School of Pharmaceutical Sciences, University of Nottingham, Nottingham NG7 2RD, UK

**Summary** 2-(4-aminophenyl)benzothiazole (CJM 126) elicits potent growth inhibition in human-derived breast carcinoma cell lines, including oestrogen receptor-positive (ER+) MCF-7$^{wt}$ cells. Analogues substituted in the 3′ position with I (DF 129), $CH_3$ (DF 203), or Cl (DF 229) possess an extended profile of antitumour activity with remarkable selective activity in cell lines derived from solid tumours associated with poor prognosis, e.g. breast, ovarian, renal and colon. Growth inhibition occurs via unknown, possibly novel mechanism(s) of action. Two cell lines have been derived from sensitive MCF-7$^{wt}$ breast cancer cells ($IC_{50}$ value < 0.001 μM) following long-term exposure to 10 nM or 10 μM CJM 126, MCF-7$^{10\ nM\ 126}$ and MCF-7$^{10\ \mu M\ 126}$ respectively, which demonstrate acquired resistance to this agent ($IC_{50}$ > 30 μM) and cross-resistance to DF 129, DF 203 and DF 229. Sensitivity to tamoxifen, benzo[a]pyrene (BP), mitomyin C, doxorubicin and actinomycin D is retained. Resistance may, in part, be conferred by the constitutively increased expression of bcl-2 and p53 proteins detected in MCF-7$^{10\ nM\ 126}$ and MCF-7$^{10\ \mu M\ 126}$ lysates. Significantly decreased depletion of CJM 126 (30 μM) from nutrient medium of MCF-7$^{10\ nM\ 126}$ cells was observed with predominantly cytoplasmic drug localization and negligible DNA strand breaks. *N*-acetyl transferase (NAT)1 and NAT2 proteins were expressed by all three MCF-7 sub-lines, but significantly higher expression of NAT2 was accompanied by enhanced acetylation efficacy in MCF-7$^{10\ nM\ 126}$ cells. In contrast, CJM 126 (30 μM) was rapidly depleted from nutrient medium of MCF-7$^{10\ \mu M\ 126}$ culture and accessed nuclei of these cells exerting damage to DNA. The major biotransformation product of CJM 126 in MCF-7$^{10\ \mu M\ 126}$ cells was 2-(4-aminophenyl)-6-hydroxybenzothiazole (6-OH 126). This metabolite possessed no antitumour activity. Accordingly, in this sub-line, low constitutive expression and activity of cytochrome P450 (CYP) 1A1 was detected. © 2000 Cancer Research Campaign

**Keywords:** 2-(4-aminophenyl)benzothiazole; MCF-7, acquired resistance

CJM 126 is an intriguing compound which emanated from a drug discovery programme initially aimed at developing tyrosine kinase inhibitors (Stevens et al, 1994). It was found to inhibit the growth of human-derived breast cancer cell lines irrespective of ER status. The unusual biphasic dose–response elicited by CJM 126 against ER+ and ER– breast cancer cell lines has been described (Bradshaw et al, 1998*a*; 1998*b*). Substitution of 2-(4-aminophenyl)benzothiazoles in the 3′ position with a halogen atom or a methyl group (Figure 1) furnished these antitumour agents with remarkable potency and selectivity (Shi et al, 1996). In vitro, $IC_{50}$ values fell below 0.01 μM in cultures of sensitive cell lines of breast (MCF-7, MDA 468, T47D), ovarian (IGROV1), renal (TK10) and colon (COLO 205, HCT 116) origin. In vivo, responsive xenograft tumours included ER+ MCF-7 and BO, ER- MaTu, MT-1 and MT-2 breast (Shi et al, 1996) as well as COLO 205 and HCT 116 colon (J Double and M. Bibby, personal communication).

Currently, selection of a clinical candidate awaits results of toxicological investigations. However, precise mechanism(s) of action remain obscure. 2-(4-Aminophenyl)benzothiazoles comprise a distinct mechanistic class of antitumour agent. Computerized pattern recognition (COMPARE) algorithm analyses (Weinstein et al, 1997) have failed to identify a biological target.

*Received 1 October 1999*
*Revised 15 February 2000*
*Accepted 19 February 2000*

*Correspondence to:* TD Bradshaw

Elucidation of biochemical mechanisms of resistance has contributed immensely to knowledge of mechanisms of anticancer drug action at the cellular and molecular level, as exemplified by methotrexate and doxorubicin (Sinha et al, 1987; Akman et al, 1990; Batist et al, 1986). Acquired drug resistance is the most common reason for failure of drug treatment in cancer patients with initially-sensitive tumours. Therefore the study of resistance mechanisms may lead to improvements in therapy following modification of drug structures such that a specific mechanism is no longer effective.

Two cell lines with acquired stable resistance to CJM 126 have been established to probe the mechanisms of action of this series

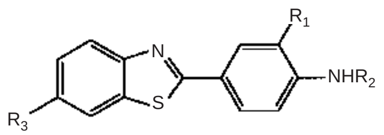

| | R¹ | R² | R³ | NSC no |
|---|---|---|---|---|
| CJM 126 | H | H | H | 34445 |
| DF 129 | I | H | H | 674496 |
| DF 203 | $CH_3$ | H | H | 674495 |
| DF229 | Cl | H | H | 682303 |
| DF 128 | H | $COCH_3$ | H | 33006 |
| 6-OH 126 | H | H | OH | 703785 |

**Figure 1** Structure of 2-(4-aminophenyl)benzothiazoles.

of agents. The variant lines, MCF-7$^{10\ nM\ 126}$ and MCF-7$^{10\ \mu M\ 126}$ were derived from MCF-7$^{wt}$ cells following prolonged exposure to 10 nM or 10 μM CJM 126 respectively.

Efforts to elucidate biochemical mechanisms conferring acquired resistance are reported herein. We have compared in the three cell lines the effects of exposure to 2-(4-aminophenyl)benzothiazoles on growth and DNA integrity. Evidence for cross-resistance to tamoxifen, BP, DNA damaging agents cisplatin, mitomycin C, the DNA intercalating agent doxorubicin and the antibiotic inhibitor of RNA synthesis actinomycin D has been studied, together with expression of genes purported to play a key role in cell survival. Depletion of CJM 126 from nutrient media, its biotransformation, intracellular distribution and the roles of xenobiotic metabolizing enzymes (NAT1, NAT2 and CYP1A1) have also been considered.

### MATERIALS AND METHODS

CJM 126, DF 129, DF 203 and DF 229 were synthesized according to published methods (Shi et al, 1996). Stock solutions of drugs (10 mM) were prepared in DMSO and stored, protected from light at 4°C for 4 weeks. RPMI 1640 tissue culture medium was obtained from Gibco (Paisley, UK). Foetal calf serum (FCS) was purchased from Globepharm (Esher, Surrey, UK). BDH (Merck, Poole, Dorset, UK) supplied fluorochrome mounting medium. NAT1 and NAT2 1° antibodies (abs), raised in rabbits were donated by Dr Lesley Stanley; specific abs directed against wt p53 (clones PAb 1801 and D-01), mutant p53 (clone PAb 240) and bcl-2 (clone 4D7) were purchased from Amersham (Bucks, UK). Anti-rabbit, anti-mouse 2° abs and reagents for enhanced chemiluminescence (ECL) detection were supplied by Pierce (Rockford, Illinois, USA). Abs (1°, 2°) specific for CYP1A1 detection, as well as the positive control sample of CYP1A1 microsomes were obtained from Gentest Corporation (Woburn, MA, USA). All other reagents were purchased from Sigma (Poole, Dorset, UK).

MCF-7 cells were cultivated in RPMI 1640 medium containing 2 μM L-glutamine and supplemented with 10% FCS, 100 IU ml$^{-1}$ penicillin and 100 mg ml$^{-1}$ streptomycin in an atmosphere of 5% $CO_2$. Cells were subcultured twice weekly to maintain logarithmic growth. Variant cell lines were derived from MCF-7$^{wt}$ following permanent culture in media containing either 10 nM or 10 μM CJM 126. Media was replaced twice weekly until proliferating colonies were established. Thereafter, MCF-7$^{10\ nM\ 126}$ and MCF-7$^{10\ \mu M\ 126}$ cell lines were routinely subcultured twice weekly in the continued presence of CJM 126.

### In vitro growth inhibitory assays

To investigate the effect of agents on growth of MCF-7 sub-lines cells were seeded into 96-well microtitre plates at densities of $5 \times 10^3$ per well. Once adhered, cells were treated with test agent; final concentration range between 0.1 nM and 100 μM (*n* = 8). MTT conversion assays, performed at the time of drug addition and following 72 h exposure, monitored cell growth and viability. Water-soluble 3-(4,5-dimethylthiazol-2-y1)-2,5-diphenyl tetrazolium bromide (MTT, final concentration 400 μg ml$^{-1}$) was added to each well. The following 4 h incubation allowed metabolism of MTT by mitochondrial dehydrogenases of viable cells to form an insoluble formazan product. Medium was aspirated and formazan solubilized by addition of DMSO (100 μl) and glycine buffer (25 μl). Absorbance, as a measure of viable cell number, was read at 550 nm on an Anthos Labtec systems plate reader. The efficacy of each agent reported was examined at least three times.

### HPLC anaysis

Medium supporting cell growth was collected (*n* = 3) and protein precipitated by addition of HPLC-grade acetonitrile (600 μl) to 300 μl medium. Samples were microcentrifuged at 13 000 rpm for 5 min and supernatant (20 μl, *n* = 2) analysed by HPLC. Separation occurred on a Hypersil ODS reversed phase column (100 mm × 4.6 mm internal diameter) using a mobile phase of 65% methanol and 35% distilled water delivered at a rate of 1 ml min$^{-1}$. Benzothiazoles and derivatives were detected by UV at 330 nm. The identities of major biotransformation products were confirmed by chromatographic analyses of authentic samples.

### Confocal laser scanning microscopy

Cells were seeded into sterile petridishes containing a single sterile coverslip in 10 ml media. They were allowed 24 h to attach before additional drug was administered to give a final concentration of 30 μM CJM 126. After 24 h exposure, cells were washed five times in sterile PBS and the coverslips drained. They were mounted onto glass slides using fluoromount and stored at 4°C in the dark. Specimens were examined using a Leica TCS4D confocal laser scanning microscope and UV laser. Images were stored on an optical disc (230 mb) and photographs produced using a Sony colour video printer. Parameters, including laser intensity, brightness and pinhole diameter were constant.

### Fluorometric analysis of DNA unwinding

The method adopted for the detection of DNA strand-breaks utilises the observation that the rate of unwinding of the two DNA strands in alkali is related to the covalent length of the strands. Applications of this sensitive and rapid technique include prediction of sensitivity to xenobiotics (Birnboim and Jevcak, 1981). The fluorescent dye ethidium bromide was used as a direct probe of DNA structure. Total fluorescence from the presence of double-stranded DNA plus contaminants was determined. Blank samples were sonicated lightly and treated with alkali under conditions which induced complete unwinding of low molecular weight double-stranded DNA. The difference between these two samples provided an estimate of the amount of double-stranded DNA. Experimental samples, including untreated cells and cells exposed to test agents, were used to estimate the rate of DNA unwinding. Cell extracts were exposed to alkaline conditions for 1 h, sufficient to permit partial unwinding of DNA. Fluorescence was read on a Perkin-Elmer LS-5 luminescence spectrometer: λem 520 nm, λex 590 nm. Samples were measured in triplicate within each experiment; experiments were performed at least three times. The rate of DNA unwinding in control and treated samples was measured, allowing agent-induced DNA strand-breaks to be determined.

### Western blot procedures

For the detection of NAT1 and NAT2, MCF-7$^{wt}$, MCF-7$^{10\ nM\ 126}$ and MCF-7$^{10\ \mu M\ 126}$ cytosolic lysates were prepared and 30 μg protein loaded onto 12% denaturing polyacrylamide gels. Electrophoresis was performed at a constant current of 0.06 A for

*© 2000 Cancer Research Campaign*

1 h. Separated proteins were transferred to polyvinylidene fluoride (PVDF) membranes (100 v, 1 h) then probed for NAT1 or NAT2 proteins. Immunoblotting with specific abs for wt or mutant p53 and bcl-2 was carried out following separation of proteins (40 µg) from whole-cell lysates on 10% gels. Incubation with 2° ab linked to horseradish peroxidase then luminol enhancer:stable peroxide (1:1) preceeded detection by ECL. Separation (10% gels) of 40 µg cellular proteins preceeded detection of CYP1A1 bands. Membranes were incubated (2 h) with goat anti-human polyclonal 1° ab (1: 500 in PBS 0.02%T, 1% milk), immersed in alkaline phosphatase conjugated rabbit anti-goat 2° ab (1: 5000, 1 h) and incubated for 10 min with BCIP and NBT substrates in alkaline phosphatase buffer.

Protein bands were subjected to densitometry using a Sharp JX330P scanner.

### Assay for ethoxyresorufin *O*-deethylase (EROD) activity

Cells were grown in 75 $cm^3$ flasks and lysates prepared following desired treatments. Protein content was determined by the method of Bradford (1976). Reaction mixtures consisting of 760 µl Tris-HCl buffer (pH 7.4, 100 mM), 10 µl $MgCl_2$ (5 mM), 100 µl homogenate (5 mg $ml^{-1}$), 100 µl ethoxyresorufin (1 mM) were pre-incubated for 5 min at 37°C. Addition of 10 µl NADPH (50 mM) initiated reactions. Blank incubates lacked NADPH. Following incubation (30 min, 37°C), reactions were terminated by addition of ice-cold acetonitrile. Samples were centrifuged at 13 000 rpm for 5 min before analysis of supernatant. Fluorescence was read on a Perkin-Elmer LS-5 luminescence spectrometer: λem 530 nm, λex 585 nm. Samples were measured in triplicate within each experiment; experiments were performed three times.

### RESULTS

### In vitro growth inhibition

The unconventional biphasic dose–response relationship following treatment of MCF-$7^{wt}$ cells with CJM 126 led to the hypothesis that the mechanism of action may be multifaceted. Thus, two variant cell lines have been established following long-term culture of MCF-$7^{wt}$ cells with: (i) a concentration of CJM 126 within the growth inhibitory phase of the dose–response curve (10 nM); (ii) a concentration within the proliferative phase of the curve (10 µM). Six months' continuous exposure to 10 nM or 10 µM CJM 126 gave rise to proliferating MCF-7 colonies which were able to resist the growth-inhibitory effects of this compound. Resistance appeared stable in vitro: following 22 passages in the absence of CJM 126 both variant cell lines revealed $IC_{50}$ values > 10 µM when rechallenged with this agent. The doubling time of MCF-$7^{10\ nM\ 126}$ did not differ significantly from the parent MCF-7 population and was approximately 24 h in log phase; slightly larger MCF-$7^{10\ \mu M\ 126}$ cells replicated in approximately 30 h. Figure 2 contrasts the dose–response profiles of MCF-$7^{wt}$ MCF-$7^{10\ nM\ 126}$ and MCF-$7^{10\ \mu M\ 126}$ to CJM 126. Following treatment for 72 h, $IC_{50}$ values of <0.001, 69.5 and 74.2 µM, respectively, were calculated. MCF-$7^{10\ nM\ 126}$ and MCF-$7^{10\ \mu M\ 126}$ cell lines demonstrated cross-resistance to DF 129, DF 203, DF 229 and DF 128 (Table 1). Sensitivity to tamoxifen, BP, doxorubicin, mitomycin C and actinomycin D was retained by MCF-$7^{10\ nM\ 126}$ and MCF-$7^{10\ \mu M\ 126}$ sub-lines. In addition, the 3 MCF-7 variant cell lines demonstrated equi-resistance to cisplatin.

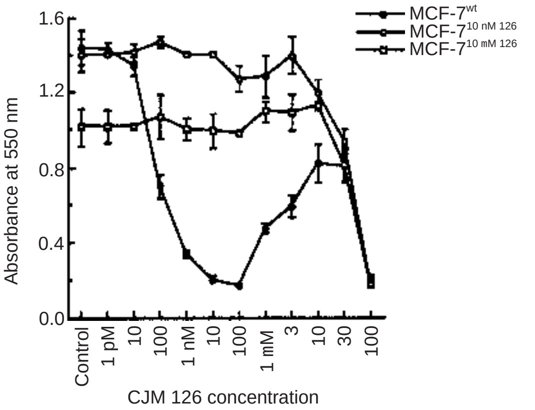

**Figure 2** Effect of CJM 126 on growth of MCF-7 populations. Cells were seeded at a density of 5 × $10^3$ per well in 96-well microtitre plates ($n$ = 8). MTT assays performed at the time of drug addition and following 72 h exposure monitored growth. Data are respresentative of experiments performed each time the stability of acquired resistance was assessed. Results are the means ± standard deviations of three experiments.

**Table 1** Effect of compounds on growth of MCF-7 populations

| | $IC_{50}$(µM) MCF-$7^{wt}$ | MCF-$7^{10nM\ 126}$ | MCF-$7^{10\ \mu M\ 126}$ |
|---|---|---|---|
| CJM 126 | <0.001 | 69.5 | 74.2 |
| DF 129 | <0.001 | 59.8 | 64.1 |
| DF 203 | <0.001 | 44.1 | 60.4 |
| DF 229 | <0.001 | 30.7 | 47.4 |
| Tamoxifen | 0.54 | 0.25 | 0.53 |
| BP | 0.314 | 0.077 | 0.097 |
| Cisplatin | >30 | >30 | >30 |
| Mitomycin C | 0.001 | 0.001 | 0.002 |
| Doxorubicin | 0.005 | 0.003 | 0.004 |
| Actinomycin D | <0.001 | <0.001 | <0.001 |
| DF 128 | 0.001 | 54.2 | 65.7 |
| 6-OH 126 | 18.2 | 10.1 | 10.4 |

Cells were seeded at a density of 5 × $10^3$ per well and challenged with agent as described in methods ($n$ = 8). MTT assays (≥ 3 per drug) following 72 h exposure assessed cell growth and viability. Mean $IC_{50}$ values are given, standard errors ≤12%

### Depletion from media of CJM 126 and its biotransformation by MCF-7 sublines

HPLC detection of CJM 126 enabled comparison of its disappearance from culture media nurturing MCF-7 cell lines. CJM 126 (30 µM) remained stable in medium alone (> 30 days at 37°C, Figure 3A) providing a standard mean peak area and indicating that drug depletion from media was a consequence of accumulation or biotransformation within cells.

CJM 126 was rapidly depleted from media sustaining MCF-$7^{wt}$ and MCF-$7^{10\ \mu M\ 126}$ cells (84% and 93% depletion in 72 h, Figure 3A). In contrast, MCF-$7^{10\ nM\ 126}$ cultures removed CJM 126 from medium at a much slower rate revealing 32% loss after 72 h incubation. All three cell lines metabolized CJM 126 (Figure 3B). HPLC chromatograms revealed distinct preferred routes of biotransformation of CJM 126 by MCF-$7^{10\ nM\ 126}$ and MCF-$7^{10\ \mu M\ 126}$ cells. Like MCF-$7^{wt}$ (Chua et al, 1999), the major biotransformation product liberated into MCF-$7^{10\ nM\ 126}$ culture medium was 2-(4-acetylaminophenyl)benzothiazole (DF 128). After 72 h incubations, calculations from mean peak areas indicated an

*© 2000 Cancer Research Campaign*

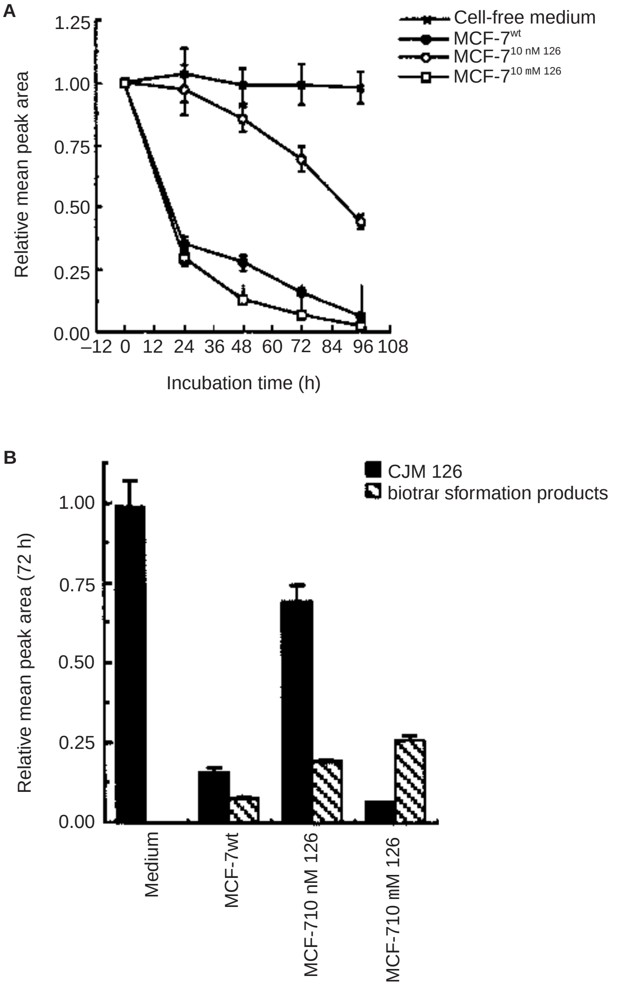

**Figure 3** Uptake and biotransformation of CJM 126 following incubation with MCF-7$^{wt}$, MCF-7$^{10nM\ 126}$ and MCF-7$^{10\mu M\ 126}$ cells. Cells were seeded at a density of $10^5$ per 25 $cm^3$ flask. Following 24 h incubation, CJM 126 was introduced (final concentration 30 μM). Media samples were removed (two per time-point) at 24 h intervals and analysed by HPLC. Means ± standard deviations of three experiments are shown. (**A**) Depletion of CJM 126 from nutrient media. (**B**) Relative levels of CJM 126 and metabolites recovered from media following 72 h drug exsposure.

approximate medium concentration of 3.7 μM DF 128. Chemically synthesized DF 128 failed to inhibit the growth of MCF-7$^{10\ nM\ 126}$ or MCF-7$^{10\ \mu M\ 126}$ cells but elicited a biphasic inhibitory dose–response in MCF-7$^{wt}$ cells ($IC_{50}$ = 0.001 μM). Minor metabolites separated included oxidized derivatives of CJM 126. In MCF-7$^{10\ \mu M\ 126}$ culture medium however, the major biotransformation product detected was 2-(4-aminophenyl)-6-hydroxybenzothiazole (6-OH 126, Figure 1). Following 72 h incubation, the approximate concentration of this metabolite detected in nutrient medium was 5 μM. Such oxidation deprived CJM 126 of growth inhibitory properties ($IC_{50}$ > 10 μM).

### Intracellular drug distribution

Fluorescent compounds may be utilised to facilitate identification of cellular sites of drug aggregation and thus provide preliminary information on intracellular targeting. Confocal microscopy, exploiting the UV fluorescence of the benzothiazole chromophore,

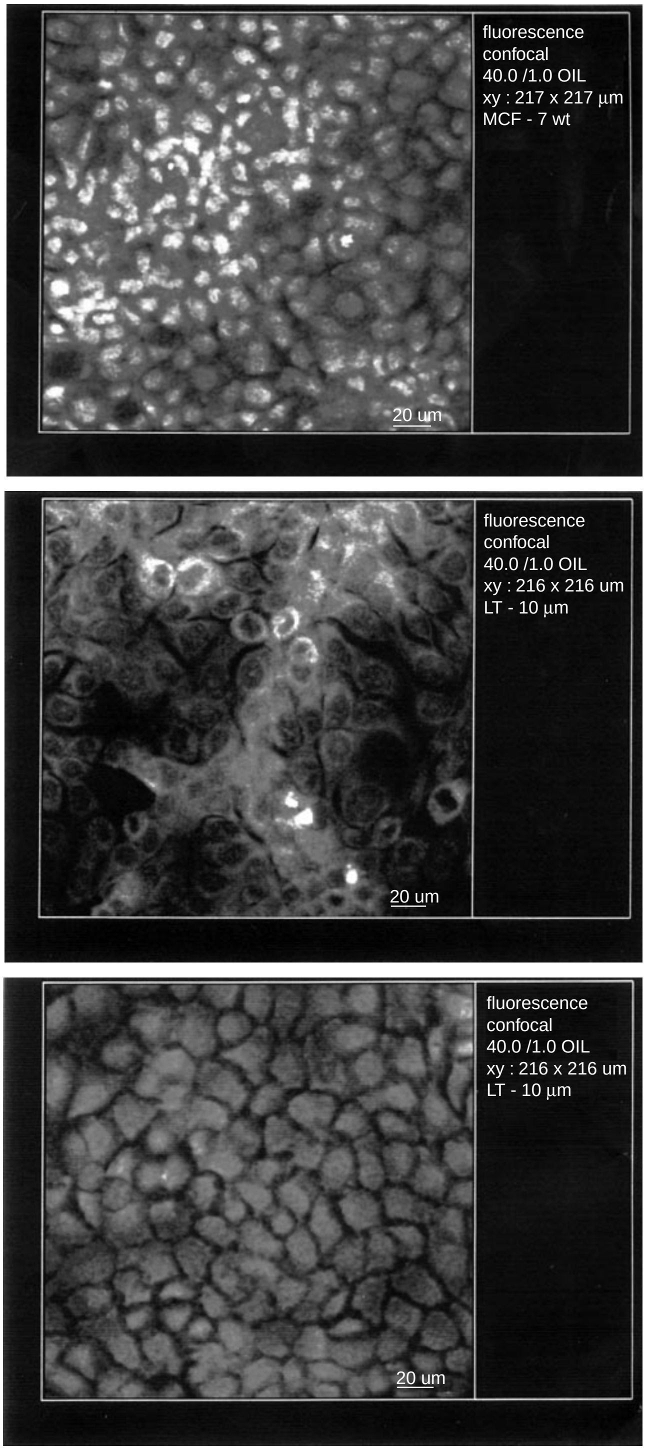

**Figure 4** Confocal laser scanning micrographs of (**A**) MCF-7$^{wt}$, (**B**) MCF-7$^{10\ nM\ 126}$ and (**C**) MCF-7$^{10\ nM\ 126}$ cells treated for 24 h with 30 μM CJM 126.

indicated differential intracellular accumulation of CJM 126 in the three MCF-7 populations following 24 h exposure to 30 μM drug (Figure 4). All fluorescence observed was drug-derived. An untreated confluent MCF-7$^{wt}$ cell monolayer observed under visible light emitted no fluorescence under UV light at the settings adopted. Fluorescence throughout MCF-7$^{wt}$ cells was observed, but particularly intense fluorescence was emitted from nuclear

*© 2000 Cancer Research Campaign* 

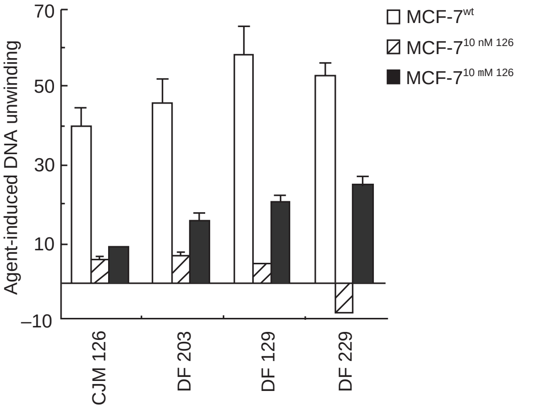

**Figure 5** Effect of benzothiazole molecules (30 µM) on DNA unwinding following 72 h exposure. DNA unwinding after 1 h was measured in MCF-7$^{wt}$, MCF-7$^{10\ nM\ 126}$ and MCF-7$^{10\ \mu M\ 126}$ cells by fluorometric analysis, enabling detection of agent-induced DNA strand-breaks. 0% represents total amount of double-stranded DNA. 100% represents complete unwinding of low molecular weight double-stranded DNA. Experiments were performed at least three times and measurements were carried out in triplicate for each experiment. Means ± standard deviations are shown.

regions. Fluorescence intensity was significantly reduced in both acquired-resistant cell lines. CJM 126 was largely restricted to the cytoplasm of MCF-7$^{10\ nM\ 126}$ cells, but accessed nuclei of MCF-7$^{10\ \mu M\ 126}$ cells.

### DNA integrity

The rate of DNA unwinding, accurately representing DNA strand-breaks accrued by MCF-7 populations, was monitored after 24 h and 72 h exposure to benzothiazole concentrations between 0.01 and 100 µM. MCF-7$^{wt}$ cells accumulated greatest damage. After 24 h treatments, the biphasic dose–response relationship (Figure 2) was reflected in rates of DNA unwinding: for example maximum strand-breaks followed exposures of 0.3 µM and ≥ 50 µM (results not shown). Figure 5 compares DNA unwinding in MCF-7 populations following 30 µM benzothiazole exposure for 72 h, prior to death of MCF-7$^{wt}$ cells. Significantly reduced strand-breaks were incurred by the two MCF-7 variant populations. In particular, damage to MCF-7$^{10\ nM\ 126}$ cells appeared negligible upon benzothiazole treatment.

### Protein expression

Western blot experiments demonstrated expression of both NAT1 and NAT2 proteins in all three MCF-7 cell lines. Significantly enhanced (> 170% compared to MCF-7$^{wt}$) expression of NAT2 protein was detected within the cytoplasm of MCF-7$^{10\ nM\ 126}$ cells (Figure 6A), consistent with their superior acetylation efficacy. CYP1A1 expression was powerfully induced in MCF-7$^{wt}$ and MCF-7$^{10\ nM\ 126}$ cells by 50 µM CJM 126 (72 h). However, constitutive expression of CYP1A1 was detected only in MCF-7$^{10\ \mu M\ 126}$ cells, whether cultured routinely in the presence of 10 µM CJM 126 (Figure 6B) or following drug absence for 14 days. Further induction by CJM 126 (50 µM, 72 h) was slight.

Substantial expression of bcl-2 protein was detected in MCF-7$^{wt}$ lysates. Up-regulation of this protein by approximately 25% was consistantly detected in MCF-7 populations with acquired resitance to CJM 126 (Figure 6A).Use of specific antibodies

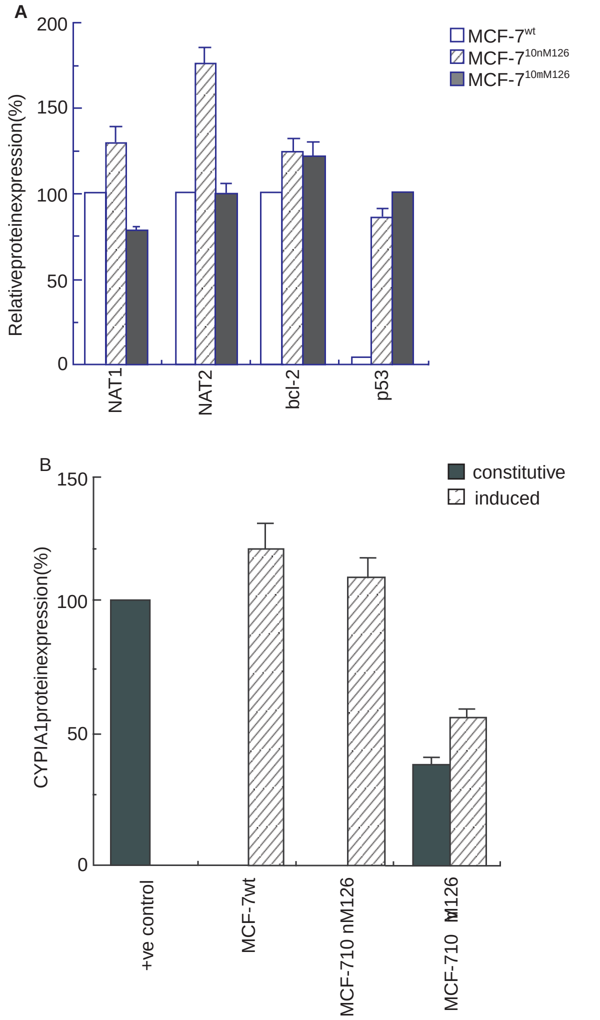

**Figure 6** (**A**) Relative expression of NAT1, NAT2, bcl-2 and wt p53 proteins in MCF-7 populations. Blots were performed at least three times from three separate sample preparations. Expression of NAT1, NAT2 and bcl-2 in MCF-7$^{wt}$ lysates represents 100%. Expression of p53 in MCF-7$^{10\ \mu M\ 126}$ lysates represents 100%. (**B**) Expression of CYP1A1 (constitutive and induced) in MCF-7$^{wt}$, MCF-7$^{10nM\ 126}$ and MCF-7$^{10nM\ 126}$ control cell lysates and following exposure of cells to 50 µM CJM 126 (72 h) respectively. Immunoblot of recombinant CYP1A1 microsomes (10 µg protein) represents the positive control, against which sample CYP1A1 expression was compared. Blots were performed twice on two separate sample preparations. Means ± standard deviations are shown.

distinguishing wt and mutant p53 demonstrated significantly enhanced expression of wt p53 in both CJM 126-resistant MCF-7 sub-lines. Protein detected in MCF-7$^{10\ nM\ 126}$ and MCF-7$^{10\ \mu M\ 126}$ lysates respectively was 23-fold and 27-fold greater than untreated MCF-7$^{wt}$ cells, which expressed very low basal levels of wt p53 (Figure 6A).

### CYP1A1 activity in MCF-7 lysates

Ethoxyresorufin *O*-de-ethylation is catalyzed by CYP1A1. CYP1A1 activity was assessed by measurement of reaction product resorufin. Negligible EROD activity was detected in untreated MCF-7$^{wt}$ lysates following 30 min incubation at 37°C. Incubates containing MCF-7$^{10\ nM\ 126}$ lysates produced low levels of resorufin. Significant constitutive CYP1A1 activity in

*© 2000 Cancer Research Campaign*

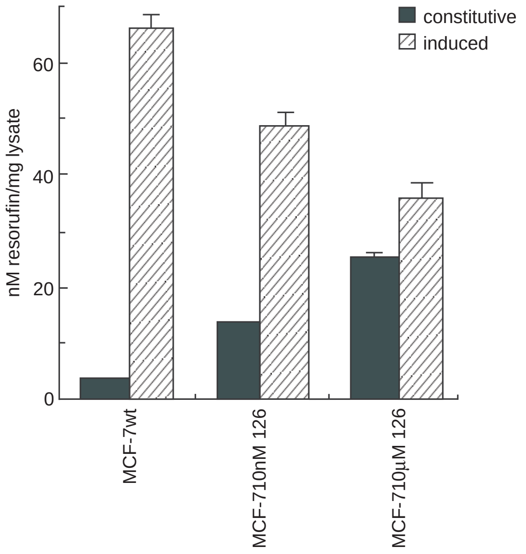

**Figure 7** Constitutive and induced *O*-de-ethylation of ethoxyresorufin by lysates of control and treated (50 µM CJM 126, 72 h) MCF-7$^{wt}$, MCF-7$^{10nM\ 126}$ and MCF-7$^{10\ \mu M\ 126}$ cells. Resorufin product was measured and means ± standard deviations of two representative experiments ($n$ = 6) are shown.

MCF-7$^{10\ \mu M\ 126}$ lysates catalysed production of 26.9 nM resorufin per mg protein. After treatment of cells with 30 µM CJM 126 for 72 h, increased EROD activity in MCF-7$^{wt}$ lysates was demonstrated by sharp rises in resorufin (> 20-fold, Figure 7). Activity was further induced in MCF-7$^{10\ nM\ 126}$ and MCF-7$^{10\ \mu M\ 126}$ incubates by 260% and 42% respectively.

### DISCUSSION

Two MCF-7 sub-lines have been established which display resistance to the prototype analogue of an intriguing and unique class of antitumour agent, the 2-(4-aminophenyl)benzothiazoles. Acquired resistance to CJM 126 conferred cross-resistance to 3′ substituted analogues (Table 1). In the NCI COMPARE program Pearson Correlation Coefficients above 0.7 were observed only between 2-(4-aminophenyl)benzothiazole analogues (Bradshaw et al, 1998*b*). Such observations indicate shared mechanisms of action. Evolution of resistance requires that sensitive cells acquire multiple genetic and biochemical modifications (Hayes and Wolf, 1990) which may be pertinent to drug action. Cognate adaptations to promote survival in the presence of 2-(4-aminophenyl)benzothiazoles have been detected in MCF-7$^{10\ nM\ 126}$ and MCF-7$^{10\ \mu M\ 126}$ cell lines. In addition, distinct mechanisms have evolved as a consequence of selection in the presence of concentrations of CJM 126 which elicit very different growth responses in MCF-7$^{wt}$ cells.

We have shown that cells lines inherently sensitive to this class of agent rapidly sequester and metabolize 2-(4-aminophenyl)benzothiazoles, whereas no net uptake was detected by intrinsically resistant cell lines (Chua et al, 1999; Kashiyama et al 1999). Compared with MCF-7$^{wt}$ and MCF-7$^{10\ \mu M\ 126}$, MCF-7$^{10\ nM\ 126}$ cells, selected in the presence of the more cytotoxic concentration of 10 nM CJM 126, demonstrated dramatically impaired depletion of CJM 126 from media (Figure 3A) with predominantly cytoplasmic intracellular drug localization (Figure 4). Accordingly, negligible DNA strand-breaks were incurred by this sub-line when challenged with 2-(4-aminophenyl)benzothiazole analogues (Figure 5) reflecting their poor sensitivity. Access to nuclei may be essential for CJM 126-induced growth inhibition in MCF-7$^{wt}$; nuclear exclusion by MCF-7$^{10\ nM\ 126}$ may aid emergence of resistance.

However, cells selected in the presence of 10 µM CJM 126 (the proliferative phase) rapidly depleted culture medium of CJM 126 (Figure 3A), accumulated CJM 126-associated fluorescence within nuclei (Figure 4) and sustained levels of DNA strand-breaks greater than MCF-7$^{10\ nM\ 126}$ but less than MCF-7$^{wt}$ (Figure 5).

The major biotransformation product of CJM 126 extruded by MCF-7$^{wt}$ cells is DF 128 (Figure 1). Activity was retained in MCF-7$^{wt}$, but with diminished potency. Cross-resistance to DF 128 was observed in both MCF-7 variant cell lines. Similarly, acetylation was the major metabolic route of CJM 126 in MCF-7$^{10\ nM\ 126}$ cells. Consistant with this observation, cytoplasmic levels of NAT1 and particularly NAT2 proteins were elevated in the MCF-7$^{10\ nM\ 126}$ sub-line.

In humans, NAT1 and NAT2 isoenzymes catalyse *N*-acetylation, *O*-acetylation and *N-O*-transacetylation of arylamines (Sadrieh et al, 1996). Two polymorphic loci on chromosome 8p22 encode these enzymes segregating individuals into fast and slow acetylator phenotypes (Stacey et al, 1996). Thus, acetylation capacity and the balance between xenobiotic activation/detoxification is subject to individual variation and, with it, the response to chemotherapeutic agents. Continued exposure of MDA 468 cells, which express only NAT1, to 10 nM or 10 µM CJM 126 has failed to generate resistant variants of this cell line, supporting a role for NAT2 in acquired resistance to CJM 126.

Biotransformation via *C*-oxidation was the preferred metabolic route of CJM 126 by MCF-7$^{10\ \mu M\ 126}$ cells; 6-OH 126 was the major metabolite rapidly detected in nutrient medium.

The role of enzymes responsible for *C*-oxidation, specifically CYP1A1 (Pelkonen and Raunio, 1997; Kress and Greenlee, 1997) has been investigated. CYP1A1 catalyzes both detoxification and activation of xenobiotics and precarcinogens (Crofts et al, 1998). Recombinant CYP1A1 catalyzed *C*-6 oxidation of the benzothiazole nucleus (Chua et al, submitted) producing metabolites which: (i) like α-naphthoflavone and apigenin (Pastrakuljic et al, 1997), inhibit CYP1A1 activity and abrogate growth inhibition induced by 2-(4-aminophenyl)benzothiazoles; (ii) like apigenin, evoke a mitogenic response between concentrations of 300 nM and 5 µM in MCF-7$^{wt}$ cells. Covalent binding between $^{14}$C labelled DF 203 and CYP1A1 further implicated this isoform as a molecular target for this class of agent. Moreover, in a panel of cell lines examined induction of CYP1A1 expression and activity by 2-(4-aminophenyl)benzothiazole was restricted to cell lines inherently sensitive to this class of agent. Significant induction by CJM 126 of CYP1A1 expression and activity was detected in MCF-7$^{wt}$ and MCF-7$^{10\ nM\ 126}$ lysates (Figure 6B and 7). However, oxidized derivatives comprised only minor biotransformation products of CJM 126 incubations with MCF-7$^{wt}$ and MCF-7$^{10\ nM\ 126}$ cells. The stable constitutive expression and activity of CYP1A1 in MCF-7$^{10\ \mu M\ 126}$ cells may equip this sub-line with the ability to biotransform potentially damaging agents to non-toxic derivatives possessing growth-promoting stimuli which may assist survival of MCF-7$^{10\ \mu M\ 126}$ cells in the presence of 2-(4-aminophenyl)benzothiazole. Alternatively, meagre inducibility of CYP1A1 expression and activity in this cell line could lead to failure in activation of

© 2000 Cancer Research Campaign 

2-(4-aminophenyl)benzothiazole and thus contribute to drug resistance. Ivy et al (1988) report low inducibility of CYP1A1 in multidrug-resistant MCF-7/Adr cells; indeed this cell line failed to respond to CJM 126 and in lysates prepared from CJM 126 treated cells, no expression of CYP1A1 protein was observed (data not shown).

The response of MCF-$7^{10\ nM\ 126}$ and MCF-$7^{10\ \mu M\ 126}$ cells to BP, the precarcinogen catalyzed to its reactive metabolite by CYP1A1, was of interest considering the implicated role of CYP1A1 in the mechanism of action of 2-(4-aminophenyl)benzothiazoles. In fact, marginally greater sensitivity was displayed by MCF-$7^{10\ nM\ 126}$ and MCF-$7^{10\ \mu M\ 126}$ cell lines indicating distinct mechanisms of resistance to CJM 126 and BP in MCF-7 sub-lines. Acquired resistance to BP was mediated by decreased activation to (+/–)-anti-benzo[a]pyrene-7, 8-diol, 9, 10-oxide (Caruso and Batist, 1999). The three MCF-7 variant lines appeared resistant to the DNA-damaging effects of cisplatin, but displayed equi-sensitivity to mitotmycin C which cross-links and alkylates DNA. MCF-$7^{10\ nM\ 126}$ and MCF-$7^{10\ \mu M\ 126}$ sub-lines also retained sensitivity to doxorubicin and actinomycin D. Comparative genomic hybridization experiments confirmed under-representation of the MDR1 region (7q21) in both resistant cell lines (L Hiorns, personal communication); inferring that acquired resistance to CJM 126 is not mediated by p-glycoprotein overexpression.

Deregulation of apoptotic machinery may serve as a novel mechanism of resistance by influencing the propensity of cells to undergo drug-induced apoptosis. In MCF-$7^{wt}$ cells exposed to 2-(4-aminophenyl)benzothiazoles, induction of wt p53 expression is concentration-dependent, biphasic and inversely proportional to growth inhibition and bcl-2 expression. Both MCF-$7^{10\ nM\ 126}$ and MCF-$7^{10\ \mu M\ 126}$ variants exhibit greatly enhanced levels of wt p53. Elevated p53 may promote survival in the presence of cytotoxins by arresting cells in the $G_1$ cell cycle phase, permitting repair of damaged DNA such that cells, rescued from the apoptotic pathway, are offered a growth advantage in the presence of the survival signal provided by enhanced bcl-2 expression (Malcomson et al, 1995; Kinzler and Vogelstein, 1994). However, sensitivity to 2-(4-aminophenyl)benzothiazoles does not depend upon functional p53 or bcl-2 expression as exemplified by the equi-sensitive human breast carcinoma cell line MDA 468. Failure of these cells, which possess mutant p53 and are bcl-2 null, to spawn acquired resistant phenotypes may signify a role for these proteins in acquired resistance.

In conclusion, it is worth stressing that anti-cancer drug resistance is a multifactorial phenomenon. Decreased intracellular drug levels (anthracyclines, vinca alkaloids and antibiotics), increased drug inactivation (alkylating agents and antimetabolites), altered expression of drug metabolizing enzymes, target enzymes or receptors (methotrexate or steroids), all exemplify strategies which may have been adopted by MCF-7 cells as they acquire resistance to 2-(4-aminophenyl)benzothiazoles.

Knowledge of acquired resistance mechanisms may serve as an important prerequisite in novel drug discovery programmes to guide rational chemical synthesis of superior analogues. Hydrogen atoms in the benzothiazole nucleus have been isosterically replaced with fluorine atoms to eliminate inactivating 6-hydroxylation and hence proliferation associated with drug exposures between 1 μM and 30 μM (unpublished). In preliminary studies, a fluorinated derivative of CJM 126 elicited nM $IC_{50}$ values in MCF-$7^{wt}$, MCF-$7^{10\ nM\ 126}$ and MCF-$7^{10\ \mu M\ 126}$ cell lines. Moreover, the second proliferative phase associated with MCF-7 exposure to CJM 126 was absent, indicating that by molecular modification, acquired resistance to CJM 126 may be circumvented.

### ACKNOWLEDGEMENTS

Part 11 of the series ‘Antitumour benzothiazoles’. This work was supported by the Cancer Research Campaign, UK. The authors thank Dr Lesley Stanley of De Montfort University for NAT1 and NAT2 primary antibodies, Andy Hubbard of the University of Leicester for confocal microscopy expertise and wish to acknowledge collaborations with the National Cancer Institute, USA and the Screening and Pharmacology Group of the European Organisation for Research and Treatment of Cancer (EORTC).

### REFERENCES

Akman SA, Forrest G, Chu FF, Esworthy S and Doroshow JH (1990) Antioxidant and xenobiotic-metabolizing enzyme gene expression in doxorubicin-resistant human breast cancer cells. *Cancer Res* **50**: 1397–1402

Batist G, Tulpules A, Sinha BK, Katki AG, Myers CE and Cowan KH (1986) Overexpression of a novel anionic glutathione transferase in multi-drug resistant human breast cancer cells. *J Biol Chem* **261**: 15544–15549

Birnboim HC and Jevcak JJ (1981) Fluorometric method for rapid detection of DNA strand breaks in human white blood cells produced by low doses of radiation. *Cancer Res* **41**: 1889–1892

Bradford MM (1976) A rapid and sensitive method for the quantitation of microgram quantities of protein utilizing the principle of protein-dye binding. *Anal Biochem* **72**: 248–254

Bradshaw TD, Wrigley S, Shi D-F, Schultz RJ, Paull KD and Stevens MFG (1998*a*) 2-(4-Aminophenyl)benzothiazoles: novel agents with selective profiles of in vitro antitumour activity. *Br J Cancer* **77**: 745–752

Bradshaw TD, Shi D-F, Schultz RJ, Paull KD, Kelland L, Wilson A, Garner C, Fiebig HH, Wrigley S and Stevens MFG (1998*b*) Influence of 2-(4-aminophenyl)benzothiazoles on growth of human ovarian carcinoma cells in vitro and in vivo. *Br J Cancer* **78**: 421–429

Caruso JA and Batist G (1999) Divergent mechanisms for loss of Ah-responsiveness in benzo[a]pyrene- and adriamycin-resistant MCF-7 cells. *Biochem Pharmacol* **57**: 1253–1263

Chua M-S, Shi D-F, Wrigley S, Bradshaw TD, Hutchinson I, Shaw PN, Barrett DA, Stanley L, Sausville EA and Stevens MFG (1999) Antitumour Benzothiazoles. 7. Synthesis of 2-(4-acylaminophenyl)benzothiazoles and investigations into the role of acetylation in the antitumor activities of the parent amines. *J Med Chem* **42**: 381–392

Chua M-S, Kashiyama E, Bradshaw TD, Stinson SF, Brantley E, Sausville EA and Stevens MFG. Role of CYPIAI in modulation of antitumour properties of the novel agent 2-(4-amino-3-methyl-phenyl) benzothiazole (DF 203, NSC 674495) in human breast cancer cells. *Cancer Research* (submitted)

Crofts FG, Sutter TR and Strickland PT (1998) Metabolism of 2-amino-1-methyl-6-phenylimdazo [4, 5-6] pyridine by human cytochrome P45501A1, P4501A2 and P4501B1. *Carcinogenesis* **19**: 1969–1973

Hayes JD and Wolf CR (1990) Molecular mechanisms of drug resistance. *Biochem J* **272**: 281–295

Ivy SP, Tulpule A, Fairchild CR, Averbuch SD, Myers CE, Nebert DW, Baird WM abd Cowan KH (1988) Altered regulation of P-4501A1 expression in a multidrug-resistant MCF-7 human breast cancer cell line. *J Biol Chem* **263**: 19119–19125

Kashiyama E, Hutchinson I, Chua M-S, Stinson SF, Phillips LR, Kaur G, Sausville EA, Bradshaw TD, Westwell AD and Stevens MFG (1999) Antitumor benzothiazoles 8. Synthesis, metabolic formation and biological properties of antitumor 2-(4-aminophenyl)benzothiazoles. *J Med Chem* **42**: 4172–4184

Kinzler KW and Vogelstein B (1994) Clinical implications of basic research: Cancer therapy meets p53. *New Engl J Med* **331**: 49–50

Kress S and Greenlee WF (1997) Cell-specific regulation of human CYP1A1 and CYP1B1 genes. *Cancer Res* **57**: 1264–1269

Malcomson RDG, Oren M, Wyllie AH and Harrison DJ (1995) p53-Independent death and p53-induced protection against apoptosis in fibroblasts treated with chemotherapeutic drugs. *Br J Cancer* **72**: 952–957

Pastrakuljic A, Tang BK, Roberts EA and Kalow W (1997) Distinction of CYP1A1 and CYP1A2 activity by selective inhibition using fluvoxamine and isosafrole. *Biochem Pharmacol* **53**: 531–538

 © 2000 Cancer Research Campaign

Pelkonen O and Raunio H (1997) Metabolic activation of toxins: tissue-specific expression and metabolism in target organs. *Env Health Perspectives* **105**: 767–774

Sadrieh N, Davis CD and Snyderwine EG (1996) *N*-acetyltransferase expression and metabolic activation of the food-derived heterocyclic amines in the human mammary gland. *Cancer Res* **56**: 2683–2687

Shi DF, Bradshaw TD, Wrigley S, McCall CJ, Lelieveld P, Fichtner I, and Stevens MFG (1996) Antitumour benzothiazoles. 3. Synthesis of 2-(4-aminophenyl)benzothiazoles and evaluation of their activities against breast cancer cell lines in vitro and in vivo. *J Med Chem* **37**: 3375–3384

Sinha BK, Katki AG, Batist G, Cowan KH and Myers CE (1987) Differential formation of hydroxyl radicals by adriamycin in sensitive and resistant MCF-7 human breast tumour cells: Implications for the mechanism of action. *Biochem* **26**: 3776–3781

Stacey M, Thygesen P, Stanley L, Matas N, Risch A and Sim E (1996) Arylamine *N*-acetyltransferase as a potential biomarker in bladder cancer: fluorescent in situ hybridization and immunohistochemistry studies. *Biomarkers* **1**: 55–61

Stevens MFG, McCall CJ, Lelieveld P, Alexander P, Richter A and Davies DE (1994) Structural studies on bioactive compounds. 23. Synthesis of polyhydroxylated 2-phenylbenzothiazoles and a comparison of their cytotoxicities and pharmacological properties with genistein and quercetin. *J Med Chem* **37**: 1689–1695

Weinstein JR, Myers TG, O'Connor PM, Friend SH, Fornace AJF, Kohn KW, Fojo T, Bates SE, Rubinstein LV, Anderson NL, Buolamwini JK, Vanosdol WW, Monks AP, Scudiero DA, Sausville EA, Zaharevitz DW, Bunow B, Viswanadhan VN, Johnson GS, Wittes RE and Paull KD (1997) An information-intensive approach to the molecular pharmacology of cancer. *Science* **275**: 343–349

*© 2000 Cancer Research Campaign*
